# Influence of the Viral Superoxide Dismutase (SOD) Homologue on Lumpy Skin Disease Virus (LSDV) Growth, Histopathology and Pathogenicity

**DOI:** 10.3390/vaccines8040664

**Published:** 2020-11-07

**Authors:** Nicola Douglass, Henry Munyanduki, Ruzaiq Omar, Sophette Gers, Paidamwoyo Mutowembwa, Livio Heath, Anna-Lise Williamson

**Affiliations:** 1 Division of Medical Virology, Department of Pathology, Faculty of Health Sciences, University of Cape Town, Cape Town 7925, South Africa; henrymunyanduki@gmail.com (H.M.); omar.ruzaiq@gmail.com (R.O.); Anna-Lise.Williamson@uct.ac.za (A.-L.W.); 2 Institute of Infectious Disease and Molecular Medicine, University of Cape Town, Cape Town 7925, South Africa; 3 The Pirbright Institute, Ash Road, Pirbright, Woking GU24 0NF, UK; 4 Pathcare VetLab, Cape Town 7463, South Africa; Sophette.gers@pathcare.org; 5 Onderstepoort Veterinary Institute, ARC, Gauteng 0110, South Africa; mutowembwap@arc.agric.za (P.M.); HeathL@arc.agric.za (L.H.)

**Keywords:** superoxide dismutase, lumpy skin disease virus, vaccine, virus–host interactions, inflammation

## Abstract

Lumpy skin disease is an important economic disease of cattle that is controlled by vaccination. This paper presents an investigation into the role of the lumpy skin disease virus (LSDV) superoxide dismutase (SOD) homologue on growth and histopathology of the virus both in vitro and in vivo. SOD homologue knock-out and knock-in recombinants (nLSDV∆SOD-UCT and nLSDVSODis-UCT, respectively) were constructed and compared to the Neethling vaccine (nLSDV) for growth in a permissive bovine cell line as well as on fertilized chick chorioallantoic membranes (CAMs). The infected CAMs were scored for histological changes. Deletion of the SOD homologue from LSDV reduced virus growth both in Madin-Darby bovine kidney (MDBK) cells as well as on CAMs. Furthermore, the knockout virus showed reduced inflammation in CAMs and more ballooning degeneration. A pilot experiment was performed in cattle to compare the lesions produced by the different LSDV constructs in the same animal. One animal developed a larger lesion to nLSDV∆SOD-UCT compared to both nLSDVSODis-UCT and nLSDV. Histological analysis of biopsies of these lesions shows less inflammation and necrosis associated with nLSDVSODis-UCT compared to nLSDV and nLSDV∆SOD-UCT. None of the vaccinated animals showed disseminated LSDV disease, indicating that the candidate vaccines are safe for further testing. Our results suggest that the SOD homologue may improve immunogenicity and reduce virulence.

## 1. Introduction

The interaction between poxviruses and their host cells is complex and involves multiple strategies of evasion both for the virus and host [1,2]. Poxviruses have evolved mechanisms of modulating cell death [3,4] and evading immune surveillance [2], targeting both extracellular cytokine signaling as well as intracellular pathways. Host responses leading to cell death are associated with cell mediated immune clearance of virus-infected cells [1] and the targeting of the cellular superoxide dismutase (SOD) enzyme is one example of the many strategies poxviruses have evolved to counteract host defenses. The Shope fibroma virus (SFV) SOD homologue has been shown to inhibit apoptosis by binding the copper chaperone for SOD (CCS) [5]. The inhibition of apoptosis is brought about by the upregulation of intracellular superoxide as a result of CCS being bound to the SFV SOD homologue and therefore preventing the transfer of copper to the cellular SOD enzyme, a requirement for superoxide dismutase activity [6]. Furthermore, a SFV SOD knockout virus was less pathogenic for rabbits compared to the wild type (wt) SFV [6]. Interestingly, the knockout virus grew to higher titers than the wt SFV in rabbit corneal fibroblast cells (SIRC cells) [6]. Similarly, a SOD homologue knockout myxoma virus recombinant showed increased growth in rabbit kidney (RK13) and baby green monkey kidney (BGMK) cells [7]. The vaccinia virus SOD homologue had no effect on virus growth in cell culture, nor did it influence virulence in mice [8].

Lumpy skin disease (LSD) has become a growing concern as an emerging disease in Europe and Asia, with the virus having spread from Africa, through the Middle East, to Turkey, Greece and, more recently, Russia and India [9,10,11]. Vaccination is the most feasible means of controlling the disease and has been shown to be successful [11]. The original Neethling vaccine was derived from passaging a field isolate 61 times in monolayers of primary lamb kidney cells followed by 20 passages on the chorioallantoic membranes of embryonated chicken eggs and then three passages in lamb kidney cell monolayers [12]. Onderstepoort Biological Products (OBP) (South Africa) produces this vaccine, referred to as nLSDV in this paper. A variant of nLSDV, called Herbivac, is produced by Deltamune (South Africa).

The first lumpy skin disease virus (LSDV) sequence to be published was that of the virulent Kenyan LSDV 2490 [13]. Subsequently, the genomic sequence of the South African vaccine strain (LSDV LW 1959) was compared to a virulent field isolate as well as LSDV 2490 [14]. A number of frame-shift mutations were found, which caused alterations to putative gene products in the form of truncations, deletions and insertions. Of note, frameshift mutations resulted in truncation of (open reading frame) ORF 131, the SOD homologue gene, as well as ORF 134, a homologue of vaccinia virus B22R. Biswas et al. [15] performed a large sequence analysis comparing field strains of capripoxvirus isolates with vaccine strains. They found the B22R-like gene homologue to be disrupted in a number of vaccine strains of capripoxvirus. The B22 homologue of monkeypoxvirus has been associated with T cell inhibition [16], and could have a similar function in capripoxviruses, explaining why the frameshift mutation/s could cause attenuation. Similarly, common ankyrin repeat proteins and kelch-like proteins were disrupted in a number of different vaccine strains [15]. The authors propose that vaccines could be improved by constructing more stable variants through genetic engineering of hotspots of mutation [15].

Herbivac was sequenced and found to have a single significant difference from nLSDV, a single nucleotide deletion, which restored the full length ORF 134 [17,18], the vaccinia virus B22R homologue. Independently, our group sequenced Herbivac and found a different single significant change from the nLSDV vaccine, in the form of a 2bp deletion in ORF 131, which restored the full-length SOD homologue [19]. The Herbivac SOD homologue is 160aa compared to the truncated 103aa for nLSDV. Due to anecdotal evidence that Herbivac was more immunogenic than nLSDV, and previous reports showing that the SOD homologue had an effect on Leporipoxvirus growth and pathogenicity [5,6], our group investigated the role of the SOD homologue on LSDV virus growth and immunopathology.

Our aim was to stabilize the SOD homologue gene by designing a gene that matched the Herbivac SOD homologue in amino acid sequence, but differed in a short region of binucleotide repeat sequences so that the gene would be less likely to mutate. This SOD homologue with improved stability is referred to as *SODis*. Initially, SOD knock-out and knock-in recombinants of LSDV were constructed, which had selection and marker genes present (nLSDV∆SOD-M, which had the SOD gene replaced by *gpt* and *eGFP*; and nLSDVSODis-M, which had the modified *SODis* as well as *gpt* and *mCherry*) [20]. nLSDV∆SOD-M and nLSDVSODis-M differed in their ability to modulate induction and inhibition of apoptosis [20]. The knock-in virus induced more apoptosis, presumably using the extrinsic pathway, yet showed higher levels of inhibition of camptothecin-induced apoptosis, suggesting that the SOD homologue inhibits the intrinsic pathway of apoptosis. Histologically, in a chick CAM model, the SOD homologue was associated with increased immune cell infiltration and mesodermal proliferation of fibroblasts [20]. The superoxide radical is known to promote cellular proliferation; and conversely, products of SOD metabolism (hydrogen peroxide) inhibit proliferation [21]. The decoy SOD in leporipoxviruses has been associated with fibromatous cell proliferation [6]. We inferred from our previous study that the increased cell proliferation and inflammation was caused by SODis [20].

The present study is an investigation into the influence of the SOD-homologue on vaccine growth and histopathology. To ensure that any differences observed are not due to the presence of foreign genes, the *gpt* and fluorescent protein reporter genes were removed from the initial constructs [20] and recombinants nLSDV∆SOD-UCT and nLSDVSODis-UCT were constructed. They were compared to one another and to nLSDV with respect to (1) growth in vitro (in bovine MDBK cells), (2) growth in vivo (in fertilized hens’ eggs), (3) histological changes in chick chorioallantoic membranes (CAMs) and (4) virulence and histological changes in cattle, the natural host for LSDV.

## 2. Materials and Methods

### 2.1. Alignment of SOD Homologues

An alignment was made of ten SOD or SOD-like protein sequences, obtained from GenBank. The protein sequence of SODis is the same as that of Herbivac [20]. Sequences aligned included full length SOD (active enzyme) from human and bovine, SOD-like ORFs from LSDV Neethling Nl 2490, LSDV Neethling vaccine LW 1959, LSDV Herbivac, myxoma virus, sheeppox virus, entomopoxvirus Amsacta moorei, vaccinia virus and part of the human copper chaperone CCS. The structures for bovine and human SOD were obtained from the protein databank (1SOS and 1EQ9). SOD alignment was completed using the TCoffee Expresso program available on the TCoffee server. Structural alignment was done on ESPript 3.0 Server.

### 2.2. Construction of nLSDV∆SOD-UCT and nLSDVSODis-UCT

The two recombinant viruses, nLSDV∆SOD-UCT and nLSDVSODis-UCT, were both constructed using nLSDV∆SOD-M [20] as a parent virus. This virus contained the marker genes gpt and eGFP in place of ORF 131, the LSDV SOD homologue. Transfer vectors—pHM1dSOD, to make nLSDV∆SOD-UCT, and pHM1-SOD_pos, to make nLSDVSODis-UCT—were constructed using standard cloning techniques, with pUC57 simple as the backbone plasmid vector. pHM1dSOD contained LSDV ORFs 130 and 132 adjacent to one another in the same orientation as that found in nLSDV. Homologous recombination with nLSDV∆SOD-M resulted in the removal of the marker genes, generating a recombinant that differed from nLSDV in the removal of ORF 131, the SOD homologue gene, only. The transfer vector pHM1-SOD_pos contained the SODis gene [20] inserted between ORFs 130 and 132, so that the recombinant generated from this transfer vector differed from nLSDV with respect to the nLSDV truncated SOD homologue being replaced with a full-length, more stable, SOD homologue, referred to as SODis. The Neethling vaccine strain (nLSDV) used in this study was obtained commercially from Onderstepoort Biological Products; it is equivalent to strain LW 1959.

#### 2.2.1. Isolation of nLSDV∆SOD-UCT

Primary lamb testes cells were seeded into a 6-well plate at 5 × 10^5^ cells/well. Within two hours of seeding, cells were infected with nLSDV∆SOD-M at a multiplicity of infection (MOI) of 0.05. Twenty-four hours later, cells were transfected with 3 µl XtremeGeneHP and 6 µg pHM1dSOD. Two days post transfection cells were freeze-thawed twice (37 °C/−80 °C), and the lysate was passaged on MDBK cells. Non-fluorescing foci were picked and passaged on MDBK cells. This was repeated for five rounds of picking, after which DNA was extracted from infected cells and used for PCR analysis. Once confirmed, the recombinant was grown from a single focus in a 96-well plate and scaled up in a hyperflask of MDBK cells, which were infected at an MOI of 0.2. As MDBK cells harbor bovine viral diarrheal virus (BVDV), the vaccine to be used for the cattle experiment was grown on chick chorioallantoic membranes (CAMs) [22].

#### 2.2.2. Isolation of nLSDVSODis-UCT

A monolayer of primary lamb testes cells was infected with nLSDV∆SOD-M (expressing eGFP) at an MOI of 0.001. Two hours post infection, the cells were transfected with 6µg of transfer vector pHM1-SOD_pos. Two days post infection and transfection cells were lysed by three cycles of freezing and thawing. The lysate was passaged on MDBK cells and three rounds of picking of non-fluorescing foci were performed. A single non-fluorescing focus was isolated on a 96-well plate and expanded in MDBK cells followed by passage on chick CAMs [22].

#### 2.2.3. PCR Confirmation of Recombinants nLSDV∆SOD-UCT and nLSDVSODis-UCT

PCR was performed to amplify the DNA between LSDV ORFs 130. The following primers were used: LSDV129 for: 5′-GAGCCCTGTAATTCACTTTT-3′ and LSDV132 for 5′-ATCGATGGAAAAGATCCG-3′. The conditions for the PCR included an initial denaturation step at 95 °C for 5 min, 25 cycles of denaturation at 95 °C for 30 s, annealing for 30 s at 59 °C and extension time at 72 °C for 3 min. A final extension time of 7 min at 72 °C was performed. Phusion^®^ High-Fidelity DNA Polymerase (New England BioLabs, Massachusetts, USA) was used. PCR products were sent to the Central Analytical Facilities at the University of Stellenbosch, South Africa, for Sanger sequencing.

### 2.3. Growth Curves in MDBK Cells

The viruses nLSDV, nLSDV∆SOD-UCT and nLSDVSODis-UCT were compared for growth in the bovine MDBK cell line. MDBK cells were seeded at a concentration of 5 × 10^5^ cells per well of a 12-well plate. Cells were infected at an MOI of 0.015 in triplicate and lysates were prepared 2 h post infection and at subsequent 1 day intervals from days 1 to 5. At each time point, a plate was frozen. Cells were frozen and thawed a total of three times before the lysate was titrated. The endpoint dilution method of Reed and Muench [23] was used to determine the virus titer. Titrations in TCID_50_ units were converted to focus forming units per ml (ffu/mL).

### 2.4. Growth Curves on Chick Chorioallantoic Membranes (CAMs)

Viruses nLSDV, nLSDV∆SOD-UCT and nLSDVSODis-UCT were compared for growth on chick CAMs. Seven-day-old specific pathogen free eggs (SPF) from Leghorn chickens (Avifarms (Pty) Ltd. (Lyttelton, South Africa)) were infected as previously described [22,24]. CAMs were inoculated with 100 µl of 10^4^ TCID_50_/_mL_ nLSDV, nLSDV∆SOD-UCT and nLSDVSODis-UCT. Eggs were incubated at 34 °C and harvested daily from day 0 to day 5. Three representative membranes were pooled per virus per time point. A low speed spin was performed (800 rpm for 1 min) and the supernatant was titrated using the TCID_50_ method [23]. Titrations were performed in triplicate.

### 2.5. Histology of Chick CAMs

A previously described method [24] was used to prepare CAMs for histology. Seven-day-old CAMs were infected with 10^3^ TCID_50_/100 µl nLSDV, nLSDVdSOD-UCT or nLSDVSODis-UCT. Membranes were harvested on days 1, 3 and 5 post infection. The membranes were fixed in 10% buffered formalin (formaldehyde (37–40%), NaH_2_PO_4_.H_2_0 (35.03 M), Na_2_HPO_4_ (anhydrous, 21.84 M)) and rolled into small cylinders, which were embedded in paraffin wax. Five sections per membrane per day were stained using hematoxylin and eosin (H&E) and evaluated using the light microscope. Blinded tissue scoring was performed on white blood cell infiltration, hyperplasia, ballooning degeneration and presence/absence of inclusion bodies in the chorionic or allantoic epithelium. Scoring was performed by a specialist veterinary pathologist Dr. Sophette Gers (PathCare VetLab, Cape Town South Africa). Ethics approval for the use of embryonated eggs was granted by the University of Cape Town, ethics number 013/016.

### 2.6. Testing of Vaccines in Cattle

All vaccines to be tested in cattle were grown on chorioallantoic membranes (CAMs) of 7-day old embryonated specific pathogen free (SPF) White Leghorn chicken eggs (AviFarms, South Africa) [22]. Viruses were tested for bovine viral diarrheal virus (BVDV) and shown to be negative. The Neethling vaccine strain was obtained from Onderstepoort Biological Products (OBP), Onderstepoort, Pretoria and Herbivac LS (Herbivac) was supplied by Deltamune, Pretoria. nLSDV∆SOD-UCT and nLSDVSODis-UCT were constructed as described above, the original LSDV backbone was nLSDV from OBP. Five animals of 6–12 months, which were LSDV naïve, were injected subcutaneously with 10^4^ TCID_50_/_mL_ of each of the four vaccines nLSDV, Herbivac, nLSDV∆SOD-UCT and nLSDVSODis-UCT on the left upper, left back, right upper and right back parts of the neck, respectively (a total of four injection sites per animal). Five animals received a PBS control inoculation. The cattle were monitored over a 14 day period for rectal temperature and lesions were measured. When circular, the diameter of the lesion was measured and if columnar, the diameter and width measured in order to calculate lesion size. On day 14, single skin biopsies (5 mm) were collected from each of the inoculation sites and stored in 10% buffered formalin. These were processed by a commercial pathology laboratory, wax-embedded, sectioned, and stained with H&E. Histopathological evaluation was performed using a light microscope by a specialist veterinary pathologist, Dr. Sophette Gers (PathCare VetLab, Cape Town South Africa). Ethics approval was granted by the ARC Onderstepoort Veterinary Institute—Animal Ethics Committee, AEC 5.18.

## 3. Results

### 3.1. Comparison of LSDV SOD Homologues to Those from Selected Poxviruses, as Well as Human and Bovine SOD Enzymes

**Figure 1:**
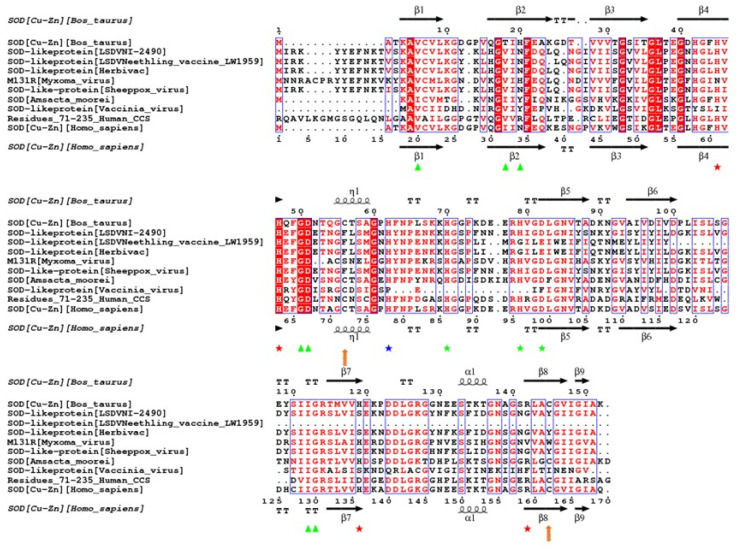
Structural alignment of selected superoxide dismutase (SOD) homologues relative to the bovine Bos taurus SOD amino acid sequence. Alignment of putative SOD-like proteins from lumpy skin disease virus (LSDV) (Neethling Nl 2490 (virulent), Neethling vaccines LW 1959 and Herbivac), with those of bovine SOD, myxoma virus, sheeppox virus, Amsacta moorei entomopoxvirus, vaccinia virus, human CCS (residues 71–235) and human SOD. The structural elements for bovine and human SOD were obtained from the protein databank (1SOS and 1EQ9). SOD alignment was done using the TCoffee Expresso program available on the TCoffee server. Structural alignment was done on ESPript 3.0 Server [29]. Residues of interest: green triangles = residues involved in dimer formation; red asterisks = copper binding residues; green asterisks = zinc binding residues; blue asterisks = residues coordinating the two metal ions; orange arrows = cysteine residues forming disulfide bridge; squiggles = α-helix and 310 helix; arrows = β-strands; TT = strict β-turns; TTT = strict α-turns.

The putative amino acid sequences of the SOD homologues from the virulent LSDV 2490 strain, the nLSDV vaccine strain and Herbivac were compared to those from selected poxviruses and the human and bovine SOD enzymes (Figure 1). The nLSDV SOD homologue (108aa) is truncated relative to LSDV 2490 (161aa) [13]. Through a frame-shift mutation, the Herbivac SOD homologue was restored to 160aa [19]. The LSDV SOD homologues show conservation of structural components (α helices and β-sheets) and the two LSDV viruses with full-length SOD homologues (LSDV 2490 and Herbivac) have retained all eight residues required for dimer formation (green triangles and blue asterisks) [25,26,27]. nLSDV, due to its truncated SOD homologue, does not have the two residues at positions 112 and 113. The three zinc binding motifs at positions 70, 79 and 82 (green asterisks) have all been conserved in LSDV 2490 and two of the three have been conserved in nLSDV and Herbivac (positions 79 and 82). Of note, two of the four copper-binding residues (positions 45 and 47) have been conserved in all LSDV SOD homologues. Similar to the Leporipoxvirus, myxoma virus, the arginine residue at position 142, which is associated with catalytic activity, is absent in all LSDV sequences (R142G). Similarly, the two cysteines at positions 56 (C56F) and 145 (C145T) are also absent in LSDV (but not myxoma virus), which are required for disulfide bridge formation. This analysis suggests that the LSDV SOD homologues may act in the same way as those of myxoma and Shope fibroma virus—as SOD decoys—which bind copper but have no superoxide dismutase activity [5,6]. The catalytic R142 and cysteines at positions 56 and 145 are preserved in all enzymes with known SOD activity, including Amsacta moorei entomopoxvirus [28]. The vaccinia virus SOD homologue lacks residues associated with enzymatic activity as well as the three zinc-binding residues, and has been shown to have no effect on virus growth or pathogenicity [8].

Genbank accession numbers are as follows: bovine SOD—NP 777040.1, LSDV Nl-2490—NP_150565.1, Neethling vaccine LW 1959—AAN02856.1, Herbivac—QBF55608.1, myxoma—NP_051845.1, sheeppox—NP_659703.1, Amsacta moorei—NP_065037.1, vaccinia A45R—YP 233053.1, residues 71–235 of human CCS—6FP6_B and human SOD—NP_000445.1.

### 3.2. Construction and Confirmation of SOD Knock-Out and Knock-In Recombinants of LSDV

**Figure 2:**
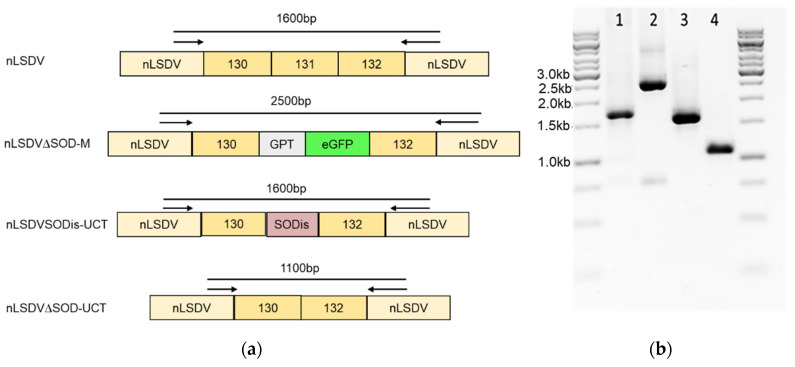
Construction of LSDV SOD knock-out and knock-in recombinants. Using a Neethling vaccine (nLSDV) backbone with the SOD homologue gene, ORF 131, replaced with *gpt* (GPT) and *eGFP* (eGFP) as the parent virus (nLSDV∆SOD-M), the two recombinants nLSDVSODis-UCT and nLSDV∆SOD-UCT were isolated. These two recombinants had no marker genes present in their genomes. (**a**) Diagrammatic representation of the recombinant viruses nLSDVSODis-UCT and nLSDV∆SOD-UCT as well as the parent virus nLSDV∆SOD-M and nLSDV, showing the sizes of anticipated PCR products using the same set of primers. (**b**) PCR products separated by 1% agarose gel electrophoresis. Lanes: 1 = nLSDVSODis-UCT, 2 = nLSDV∆SOD-M, 3 = nLSDV, 4 = nLSDV∆SOD-UCT.

To investigate the effect of the SOD homologue on growth and immunogenicity of LSDV, SOD knock-out and knock-in recombinants were constructed. The SOD homologue for the knock-in recombinant was as previously described, with the amino acid sequence identical to that of the Herbivac SOD homologue, but with an altered nucleotide sequence to reduce the chance of back mutation to a truncated SOD as is found in nLSDV [20]. Figure 2 shows PCR confirmation of these constructs, the DNA sequences of these products were confirmed to be correct by Sanger sequencing. The two recombinants, nLSDV∆SOD-UCT and nLSDVSODis-UCT, were compared biologically to determine the effect of the SOD homologue on virus growth and histopathology.

### 3.3. Growth Comparisons of nLSDV∆SOD-UCT and nLSDVSODis-UCT In Vitro and In Vivo

**Figure 3:**
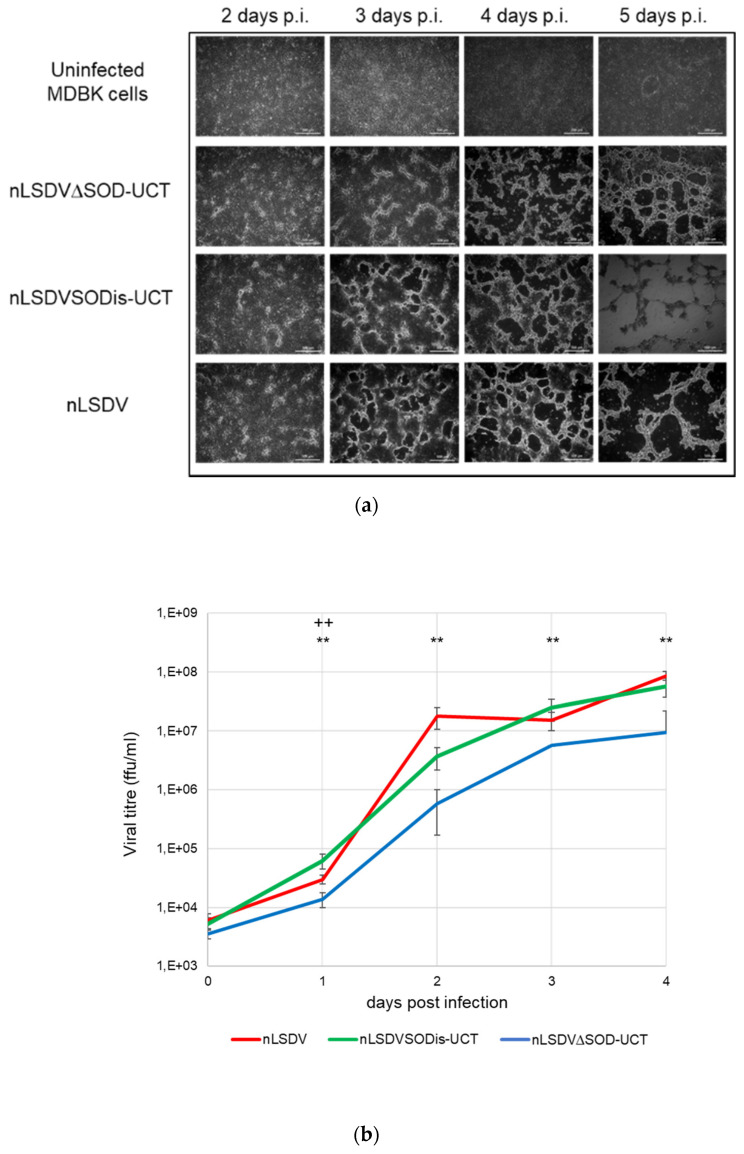
Growth in MDBK cells (**a**) MDBK cells uninfected or infected with nLSDV∆SOD-UCT, nLSDVSODis-UCT or nLSDV at an multiplicity of infection (MOI) of 0.015. Monochrome light inverted microscopic images were taken at time points as indicated; (**b**) growth curves of nLSDV (red), nLSDVSODis-UCT (green) and nLSDV∆SOD-UCT (blue) in MDBK cells. Cells were infected at a MOI of 0.015 in triplicate and lysates were prepared 2 h post infection and at subsequent 1 day intervals. Titrations were performed to determine the TCID50 in MDBK cells; titers were converted to focus forming units per ml (ffu/mL). Data were analyzed by one-way ANOVA and post-hoc T-test. Data points denote mean and error bars SEM. ** denotes significant difference between nLSDV and nLSDV∆SOD-UCT at *p* < 0.01; ++ denotes significant difference between nLSDV∆SOD-UCT and nLSDVSODis-UCT at *p* < 0.01.

**Figure 4:**
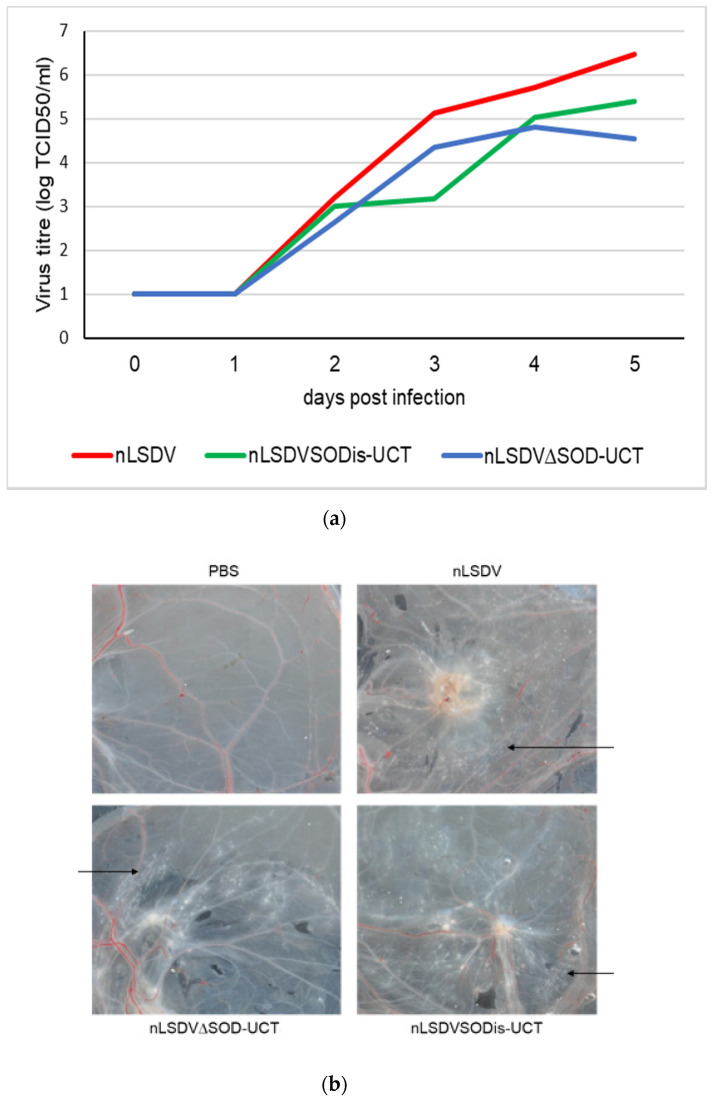
Growth in fertilized 7 day old hens’ eggs. (**a**) Growth curves of nLSDV, nLSDV∆SOD-UCT and nLSDVSODis-UCT on chick chorioallantoic membranes (CAMs) of fertilized hens’ eggs. First, 7 day-old fertilized hens’ eggs were infected with virus at 10^3^ TCID50 per egg and incubated at 34 °C. Eggs were harvested daily from days 0 to 5, triplicate membranes were pooled, and crude virus extracts were titrated in triplicate. The data shown are mean values of titers, expressed as TCID50/mL. The triplicate values were too close to show error bars. (**b**) Macroscopic appearance of infected CAMs on day 5 post infection. Representative images are shown. Black arrows point towards pocks.

nLSDV∆SOD-UCT and nLSDVSODis-UCT were compared for growth in MDBK cells (Figure 3), as well as on fertilized chick chorioallantoic membranes (CAMs) (Figure 4). Although the cytopathic effects (CPE) produced by the two viruses were similar in MDBK cells, nLSDVSODis showed greater CPE at five days post infection (Figure 3a). The growth curve of Figure 3b shows nLSDV∆SOD-UCT to have almost 10-fold reduction in growth after 4 days compared to both nLSDV and nLSDVSODis-UCT. Similarly, when fertilized hens’ eggs were inoculated with the same amount of virus, nLSDV∆SOD-UCT grew to a significantly lower titer than both nLSDV and LSDVSODis-UCT at five days post infection (Figure 4a). nLSDV produced a curve similar to that of nLSDVSODis-UCT but grew to higher titers at all time points. Macroscopically, no difference could be observed between the pocks produced by the three different LSDV viruses (Figure 4b).

### 3.4. Histological Comparisons of Chick CAMs Infected with nLSDV∆SOD-UCT and nLSDVSODis-UCT

**Figure 5:**
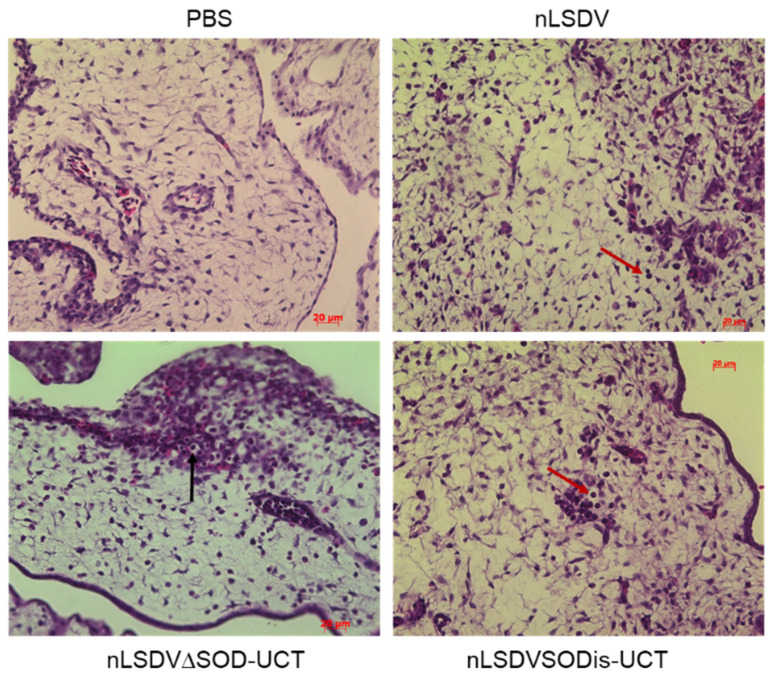
Histological analysis of infected CAMs. Hematoxylin and eosin (H&E) staining of fixed membranes 5 days post inoculation with PBS, nLSDV, nLSDV∆SOD-UCT and nLSDVSODis-UCT. Arrows indicate ballooning degeneration (black arrow) and white blood cell infiltration (red arrow); 400× magnification.

For all virus-infected membranes, inflammation (oedema, hemorrhage and white blood cell infiltration) was observed and found to be localized to the mesoderm. The grade of inflammation progressively increased from day one to day five. This was associated with hyperplastic changes, primarily in the chorionic epithelium, and the presence of typical poxvirus inclusion bodies (swollen spherical, eosinophilic, intracytoplasmic inclusions) (Figure 5). In general, membranes infected with nLSDV and nLSDVSODis-UCT achieved higher scores for each time point than those infected with nLSDV∆SOD-UCT. nLSDVSODis-UCT produced more intracytoplasmic viral inclusions than nLSDV and nLSDV∆SOD-UCT, whereas nLSDV and nLSDV∆SOD-UCT induced more vacuolar changes associated with the inclusions. Table 1 shows the scores at 5 days post infection.

### 3.5. The Effect of the LSDV SOD Homologue in Its Natural Bovine Host

**Figure 6:**
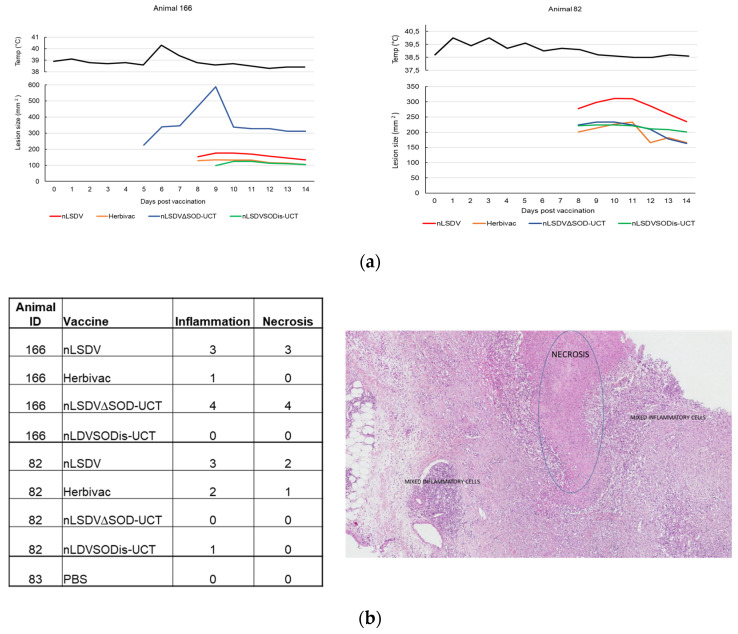
Lesions produced in cattle and histological scoring of biopsies at 14 days post infection. A group of five Friesian cattle (6–12 months old) were each inoculated with the four different LSDV vaccines, nLSDV, Herbivac, nLSDV∆SOD-UCT and nLSDVSODis-UCT, at a dose of 10^4^ TCID50 for each vaccine and monitored daily for temperature and lesion size. (**a**) Rectal temperatures of animals 166 and 82 and surface area measurements of lesions induced by nLSDV, Herbivac, nLSDV∆SOD-UCT and nLSDVSODis-UCT. (**b**) Histological scoring of biopsies taken from the lesions of animals 166 and 82. As a negative control, a biopsy was also taken from the site of PBS inoculation of animal 83. The image shows the typical appearance of regions of necrosis and inflammation.

A small pilot experiment was performed in cattle to investigate what effect the LSDV SOD homologue would have in its natural host. Of the five cattle, each inoculated with four different LSDV vaccines (nLSDV, Herbivac, nLSDV∆SOD-UCT and nLSDVSODis-UCT), only two developed lesions at the sites of inoculation. These two animals (166 and 82) both developed lesions to all four vaccines (the other three animals developed no lesions at all). In animal 166, nLSDV∆SOD-UCT produced a lesion 5 days post infection (p.i.), which became noticeably enlarged by day nine p.i. In comparison, nLSDVSODis-UCT only developed a lesion at day nine p.i. and this lesion was of comparable size to those produced by nLSDV and Herbivac, which both produced lesions at day eight p.i. (Figure 6a). For animal 82, the three vaccines Herbivac, nLSDV∆SOD-UCT and nLSDVSODis-UCT all produced lesions of similar sizes, all developing at 8 days p.i. nLSDV produced a larger lesion, also at day eight p.i. (Figure 6a). Both these animals developed high titers of neutralizing antibodies (1:64 for animal 166 and 1:128 for animal 82). Two of the animals that did not respond had relatively low titers (1:8 and 1:16) and one animal had a negative neutralization response. All animals had no demonstrable neutralizing antibodies at the start of the experiment.

At day 14 post infection, all lesions were biopsied and evaluated histopathologically following H&E staining (Figure 6b). Localized areas of necrosis, accompanied by inflammatory cells, were observed in the panniculus. This change was characterized by loss of cellular detail, fibrin exudation and infiltration of eosinophils, neutrophils and histiocytes. The inflammatory cells were often arranged in a perivascular and perilymphatic manner; rare fibrinous thrombi were also noted. Interestingly, for animal 166, the larger lesion size produced by nLSDV∆SOD-UCT was associated with higher scores of inflammation and necrosis. In contrast, nLSDVSODis-UCT showed no signs of inflammation or necrosis by day 14. Herbivac, which encodes a SOD homologue of the same amino sequence as that of nLSDVSODis-UCT, also showed less inflammation and necrosis compared to both nLSDV and nLSDV∆SOD-UCT in animal 166. This pattern of histopathology was not observed in animal 82; however, the lesion produced by nLSDV∆SOD-UCT was noticeable smaller by day 14 p.i. By comparison, the lesion produced by nLSDV was larger and gave a higher score for inflammation and necrosis.

## 4. Discussion

An investigation was made into the effect that the SOD homologue could have on LSDV growth, both in vitro and in vivo, and on host cell pathology, using the chick CAM model as well as the natural LSDV host, cattle. Clear differences were observed between the two viruses nLSDVSODis-UCT and nLSDV∆SOD-UCT, indicating a role for the SOD homologue in the life cycle and possibly pathogenesis of the virus.

An alignment of selected SOD homologue sequences confirmed the conservation of structural features in putative LSDV SOD homologues, including two of the four copper binding residues. The lack of a key catalytic site, and similarities to the Leporipoxvirus SOD homologues, point towards the LSDV SOD homologues functioning as host SOD decoys, similar to those of the Leporipoxvirus homologues [5,6,7].

Deletion of the SOD homologue from nLSDV caused a reduction in viral growth, both in MDBK cells as well as on chick CAMs. This is in contrast to what was observed for leporipoxviruses, where deletion of the SOD homologue from SFV and myxoma virus resulted in increased growth in SIRC cells and RK13 cells, respectively [6,7]. Poxviruses are known to evade cell death, which is induced by viral infection of the host cell [3,4]. It is not clear how the SOD homologue influences the final balance between induction and inhibition of cell death as it was previously shown that the Herbivac and SODis SOD homologues both induce apoptosis (presumably via the extrinsic pathway) and inhibit camptothecin-induced apoptosis (intrinsic pathway) [20]. The reduced growth of nLSDV∆SOD-UCT in cells could be attributed to the lack of inhibition of the intrinsic pathway of apoptosis, which would lead to a stronger host apoptotic response to the virus infection. In the CAM model, the kinetics of growth appeared to be different for nLSDVSODis-UCT. A possible explanation is that there is an initial increase in apoptosis via the extrinsic pathway, followed by inhibition of the intrinsic pathway of apoptosis. The later inhibition of apoptosis would favor increased viral growth of nLSDVSODis-UCT compared to nLSDV∆SOD-UCT. Interestingly, nLSDV exhibited similar growth kinetics to nLSDV∆SOD-UCT yet grew to a higher titer than nLSDVSODis-UCT. We speculate that the truncated SOD homologue of LSDV has retained certain functional activity to influence viral growth and may have contributed towards the improved growth of nLSDV during its long history of passaging in cell culture and in eggs [12]. The putative truncated protein does possess two of the four copper binding residues as well as many structural features of SOD homologues.

LSDV completes its replication cycle in eggs. Histological evaluation of inflammation in infected chick CAMs showed reduced inflammation associated with the deletion of the SOD homologue in nLSDV∆SOD-UCT. This—more quantitative analysis—confirmed what was previously observed when histological analysis was performed on CAMs infected with the SOD knock-out and knock-in viruses, which had marker genes present in their genomes [20]. We hypothesize that the incorporation of a SOD homologue into candidate poxvirus vectors would improve the immunogenicity of a vaccine by stimulating the inflammatory response.

In the pilot experiment, all four vaccines were tested on the same animal because it is known that the genetic background of cattle results in different reaction rates and so a very large number of animals are needed to compare the “take” of different vaccines if tested individually. Our experiment showed that only two out of the five animals inoculated with four different vaccines produced lesions at the sites of inoculation. However, four of the five had neutralizing antibodies. This is higher than that reported in a trial in Belgium, where 50% of cattle inoculated with nLSDV had antibodies, but all were protected from virulent LSDV challenge [29]. In one of the two animals, the lesion produced by nLSDV∆SOD-UCT was significantly larger than those produced by the other vaccines. Histological evaluation completed at 14 days post infection showed a marked increase in inflammation and necrosis associated with nLSDV∆SOD-UCT (scores of four) as compared to nLSDVSODis-UCT and Herbivac, which showed almost no effect at this time. nLSDV also produced a strong inflammatory response and necrosis (scores of three). Although any conclusion drawn from this single animal should be taken with caution, a possibly explanation for our observation could be that deletion of the SOD homologue reduces the clearance of the virus, leading to a larger lesion, and the presence of the SODis gene may contribute towards an early inflammatory response, which clears the virus infection more effectively. Although this small pilot experiment in cattle shows that none of the vaccines caused multiple/disseminated lesions, a larger cattle experiment is required to determine whether the deletion of the SOD homologue from nLSDV may increase its virulence. This experiment does, however, show that the nLSDVSODis-UCT vaccine is safe in cattle and no more virulent than the present commercial vaccines nLSDV and Herbivac. It is also noteworthy that nLSDVSODis-UCT grew to higher titers than nLSDV∆SOD-UCT in tissue culture and eggs, which is a desirable trait for a potential vaccine.

To determine whether the SOD homologue does indeed improve immunogenicity, additional experiments are required, whereby nLSDV∆SOD-UCT and nLSDVSODis-UCT should be tested in a larger cattle experiment, where separate groups of animals are infected with the two different vaccines and tested for immunogenicity against LSDV. If the SOD homologue is indeed advantageous in a vaccine, then LSDVSODis-UCT may be an improved nLSDV vaccine in that it is more genetically stable in the SOD homologue locus than either nLSDV or Herbivac.

## 5. Conclusions

Two genetically modified viruses, based on the Neethling strain of LSDV (nLSDV), have been constructed and compared to the parent nLSDV. These potential vaccines were modified at the SOD homologue locus; nLSDV∆SOD-UCT having the SOD homologue gene deleted and nLSDVSODis-UCT having the SOD homologue gene replaced with a full-length SOD homologue gene, genetically optimized to reduce mutation in a run of binucleotide repeats. The encoded amino acid sequence of the SODis gene is identical to that of the Herbivac SOD homologue. Deletion of the SOD homologue reduced viral growth in MDBK cells as well as on fertilized chick CAMs. This was also associated with reduced inflammatory changes in the mesoderm of infected chick CAMs. A preliminary experiment in cattle showed that neither of the recombinant vaccines caused disseminated disease and, in one animal, the full-length SOD homologue was associated with reduced necrosis and inflammatory responses at day 14 post infection. These findings justify further research into the inclusion of a full-length SOD homologue gene into future potential LSDV vaccines and recombinants thereof.

## 6. Patents

This work resulted in a patent being filed on the 17 May 2019, international application number PCT/IB2019/054090 (agency reference PA166012PCT)

## Figures and Tables

**Table 1:** Histology. Chorioallantoic membranes (CAMs) were inoculated with PBS, nLSDV, nLSDVdSOD-UCT and nLSDVSODis-UCT. Five days post infection, the CAMs were formalin fixed, and paraffin embedded sections were stained with H&E. Scoring was based on presence of intracytoplasmic inclusions, epithelial hyperplasia, and inflammatory changes.

Chorioallantoic Membranes: Histological Changes						
Sample	Section	Intracytoplasmic Inclusions	Epithelial Hyperplasia		Inflammatory Changes	Additional Comments
			Chorionic	Allantoic	Mesoderm	
nLSDV	A	+	4	2	5	ballooning and inclusions
	B	+	4	1	4	
	C	+	4	1	5	
	D	+	5	1	5	
	E	+	4	1	5	
nLSDV∆SOD-UCT	A	+	2	1	3	
	B	+	3	2	3	ballooning degeneration
	C	+	3	2	2	ballooning degeneration
	D	+	3	1	3	
	E	+	3	2	3	
nLSDVSODis-UCT	A	+	4	1	5	
	B	+	4	1	4	
	C	+	2	0	3	
	D	+	3	0	4	
	E	+	4	1	5	high numbers of inclusions (chorion)
PBS	A	−	0	0	0	
	B	NP	NP	NP	NP	
	C	NP	NP	NP	NP	
	D	NP	NP	NP	NP	
	E	NP	NP	NP	NP	

0—not detectable; 1—minimal (barely detectable in a minority of fields); 2—mild (barely detectable, but present in most fields); 3—moderate (clearly visible in at least 50% of fields); 4—marked (clearly visible in more than 50% of fields); 5—severe (outspoken changes in the majority of fields); NP (not performed). Viral inclusions in at least one field are scored + and − indicates no detection of viral inclusion bodies.
